# Cardioprotective Properties of Phenolic Compounds: A Role for Biological Rhythms

**DOI:** 10.1002/mnfr.202100990

**Published:** 2022-03-28

**Authors:** Cristina Torres‐Fuentes, Manuel Suárez, Gerard Aragonès, Miquel Mulero, Javier Ávila‐Román, Anna Arola‐Arnal, Maria Josepa Salvadó, Lluís Arola, Francisca Isabel Bravo, Begoña Muguerza

**Affiliations:** ^1^ Nutrigenomics Research Group Departament de Bioquímica i Biotecnologia Universitat Rovira i Virgili Tarragona 43007 Spain

**Keywords:** cardiovascular diseases, circadian rhythms, endothelial function, oxidative stress, polyphenols

## Abstract

Cardiovascular diseases (CVD) are the leading cause of deaths worldwide and their prevalence is continuously increasing. Available treatments may present several side effects and therefore the development of new safer therapeutics is of interest. Phenolic compounds have shown several cardioprotective properties helpful in reducing different CVD risk factors such as inflammation, elevated blood pressure, hyperlipidemia, or endothelial dysfunction. These factors are significantly influenced by biological rhythms which are in fact emerging as key modulators of important metabolic and physiological processes. Thus, increased events of CVD have been observed under circadian rhythm disruption or in winter versus other seasons. These rhythms can also affect the functionality of phenolic compounds. Indeed, different effects have been observed depending on the administration time or under different photoperiods. Therefore, in this review the focus will be on the potential of phenolic compounds as therapeutics to prevent CVD via biological rhythm modulation.

## Graphical abstract

Phenolic compounds exert cardioprotective properties improving oxidative stress, inflammation, and endothelial dysfunction. All of them are modulated by biological rhythms, whose disruption also boosts cardiovascular diseases. The cardiovascular properties of phenolic compounds may be mediated by biological rhythms.

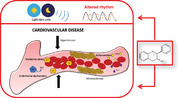

## 1 Introduction

Cardiovascular diseases (CVD) are a group of disorders that include coronary heart disease, cerebrovascular disease, peripheral arterial disease, deep vein thrombosis, and pulmonary embolism, among others.^[^ ^1^ ^]^ CVD remain the leading cause of disease burden and of death globally regardless of race, ethnicity, or sex.^[^ ^2^ ^]^ Thus, the American Heart Association reported 800 000 new cases of heart failure from 2012 to 2017.^[^ ^3^ ^]^ According to the World Health Organization 17.9 million people died from CVD in 2019, representing 32% of all global deaths. Unhealthy lifestyles including overeating, alcohol consumption, smoking and low level of physical activity are among the main behavior risk factors for CVD.^[^ ^2^ ^]^ Implementation of healthy lifestyle, including eating more fruits and vegetables and increase physical activity is the most recurrent method to reduce CVD risk in these patients.^[^ ^4^, ^5^ ^]^ Antihypertensive, antidiabetic, and lipid‐lowering drugs are useful to prevent CVD but they may present several side effects such as hypotension, dizziness, or hyperkalemia, among others.^[^ ^6^ ^]^ Therefore, there is a need to develop new therapeutic treatments with reduced side effects and suitable for patients at risk of CVD.

Phenolic compounds are one of the main phytochemicals that are produced under stress conditions and involve thousands of structurally different molecules.^[^ ^7^ ^]^ They have been shown to exert several beneficial health effects in different chronic diseases including CVD. Thus, phenolic compounds can be helpful in reducing different CVD risk factors such as inflammation, elevated blood pressure (BP), hyperlipidemia, or endothelial dysfunction.^[^ ^8^, ^9^ ^]^ On the other hand, these physiologic and metabolic processes are significantly influenced by biological rhythms.^[^ ^10^ ^]^ Hence, alterations in these rhythms may lead to changes in the cardiovascular system promoting the development of CVD. Indeed, the incidence of stroke, myocardial infarction, and sudden cardiac death show circadian patterns, being more frequent in the morning.^[^ ^11^ ^]^ In addition to circadian rhythms, many studies have reported increased frequency of CVD‐related hospitalizations and mortality in winter, which is probably influenced by circannual rhythms, depending on environmental factors such as temperature and day‐time length.^[^ ^12^ ^]^ Therefore, biological rhythms should be considered in the study of the potential of polyphenols to prevent the development of metabolic diseases such as CVD. Indeed, the activity of bioactive compounds is significantly influenced by biological rhythms. Recently, we discussed that their extensive activity could be explained by their potential to stimulate homeostasis through interactions with the biological clock system.^[^ ^13^ ^]^ Biological rhythms have been shown to impact phenolic compounds absorption and metabolism, influencing their bioactivity and, at the same time, phenolic compounds may modulate clock genes and related signaling pathways. This is in accordance with the xenohormesis hypothesis, which proposes that ingestion of phytochemicals, including phenolic compounds, allows mammals to adapt their metabolism and physiology to changes in their environment conditions.^[^ ^14^ ^]^

Hence, in this review we will focus on the potential of phenolic compounds as therapeutic for CVD and how biological rhythms influence their cardiometabolic properties. Moreover, the role of these rhythms in the different physiological and metabolic processes that contribute to the development of this cluster of diseases will be also discussed.

## 2 Molecular Mechanisms Involved in the Cardioprotective Properties of Phenolic Compounds

Evidence shows that certain phenolic compounds can be helpful in decreasing CVD risk factors.^[^ ^8^, ^15^, ^16^, ^17^ ^]^ Their cardiovascular protective effects are mainly based on its capabilities of reducing inflammatory signaling and endothelial dysfunction and improving lipid profile, oxidative stress, sirtuin (SIRT) activity, survival signaling, and calcium homeostasis.^[^ ^18^ ^]^

Atherosclerotic plaque formation is the main condition that underlies cardiometabolic diseases and is defined as a chronic inflammatory and pro‐oxidative molecular condition.^[^ ^19^ ^]^ This may suffer an erosion or get broken leading to clinical events such as myocardial infarction or angina.^[^ ^19^ ^]^ Atherosclerotic progression is characterized by increased levels of circulating tumour necrosis factor (TNF)‐α, soluble interleukin (IL)‐2 receptors, IL‐6, C reactive protein and cholesterol.^[^ ^20^ ^]^ Several studies demonstrated that phenolic compounds reduce atherosclerotic plaque formation. Resveratrol was able to counteract the overexpression of the vascular and intracellular adhesion molecules 1 (*VCAM1* and *ICAM1*) in endothelial cells through its inhibitory effect on the nuclear factor kappa B (NF‐κB) pathway.^[^ ^21^ ^]^ Interestingly, quercetin decreased mRNA and protein levels of TNF‐α, IL‐6, macrophage inflammatory protein 1‐α, and P‐selectin in murine RAW264.7 macrophage cells.^[^ ^22^ ^]^ Furthermore, 4′‐methoxyresveratrol reduced the advanced glycation end products‐induced inflammation via nuclear factor kappa‐light‐chain‐enhancer of activated B cells (NF‐κB) pathway.^[^ ^23^ ^]^ In addition, oral administration of resveratrol and different flavonoid mixtures have been shown to induce SIRT1‐mediated NF‐κB inhibition.^[^ ^24^, ^25^ ^]^ Acetylation of lysine 310 of RelA/p65 NF‐κB subunit stimulates proinflammatory responses. Consequently, the diminution of this acetylation level induced by SIRT1 promotes anti‐inflammatory effects. In addition to pure phenolic compounds, red wine has also demonstrated anti‐inflammatory properties, decreasing plasmatic concentrations of IL‐6 and ICAM‐1 as well as the expression of monocyte and T‐lymphocyte markers in comparison to beverages with low polyphenols content.^[^ ^26^ ^]^

Phenolic compounds have also been widely demonstrated to have a hypolipidemic effect in different in vivo studies. Circulating lipids such as cholesterol, apolipoprotein B‐containing lipoproteins, and triglycerides significantly contribute to atherosclerosis and CVD.^[^ ^27^ ^]^ Acute oral dose of a grape seed proanthocyanidin extract (GSPE) reduced plasma triglycerides and apolipoprotein B concentrations and significantly improved the atherosclerotic risk index in healthy rats.^[^ ^28^ ^]^ In addition, a chronic treatment with GSPE also corrected dyslipidemia associated with high‐fat diet‐induced obese rats.^[^ ^29^ ^]^ Interestingly, it has been suggested that proanthocyanidins induce hypolipidemia by reducing lipoprotein secretion, and not by increasing lipoprotein clearance.^[^ ^30^ ^]^ Reduced gut absorption of dietary fat, diminished chylomicrons secretion^[^ ^31^ ^]^ and repression of liver VLDL secretion also appears to play an important role in reducing plasma lipids concentrations.^[^ ^32^ ^]^ Proanthocyanidins also activate the Farnesoid X receptor (FXR), upregulating the *nuclear receptor small heterodimer partner* and, consequently, repressing the *sterol regulatory element‐binding transcription factor 1* (*Srebp1)* in liver. Furthermore, proanthocyanidins modulate cholesterol homeostasis through the repression of miR‐33 that, in turn, activate *ATP‐binding cassette subfamily A member 1 (Abca1)* gene expression, thus increasing HDL formation and the reverse transport of cholesterol for its elimination in the liver.^[^ ^33^ ^]^

Phenolic compounds are also known for their antioxidant properties.^[^ ^34^ ^]^ Thus, one of the main mechanisms involved in the vasoprotective properties of polyphenols is activating endogenous antioxidants.^[^ ^35^ ^]^ Human studies also support these beneficial effects. Virgin olive oil consumption for 3 weeks decreased the plasmatic concentrations of oxidized LDL, conjugated dienes, and hydroxyl fatty acids.^[^ ^36^ ^]^ Evidences show that the main effect of phenolic compounds over oxidative stress could be largely explained by its antioxidant capacity as well as its ability to increase the activity of different oxygen‐free radical scavenging enzymes.^[^ ^37^ ^]^ For example, resveratrol is very effective against in vitro oxidation of LDL^[^ ^38^ ^]^ and also induces the expression, through a nuclear factor erytheroid‐derived‐2‐like 2 (NRF2) mechanism, of superoxide dismutase (SOD), glutathione peroxidase (GPx) and catalase (CAT) in cardiac and aortic smooth muscle cells.^[^ ^39^ ^]^ Moreover, hypertensive rodents administered resveratrol showed increased activity of SOD,^[^ ^37^, ^40^, ^41^ ^]^ GPx,^[^ ^41^ ^]^ and CAT.^[^ ^40^ ^]^ In addition, quercetin regulates enzymes such as hemeoxygenase‐1 (HO‐1)^[^ ^42^ ^]^ or aortic NADPH oxidase (NOX) and reduces *Nox4* expression in aorta. Remarkably, NOX4 is the main reactive oxygen species (ROS) producer in the endothelium.^[^ ^43^ ^]^ Moreover, quercetin affects oxidative stress by inhibiting myeloperoxidase, significantly decreasing LDL oxidation.^[^ ^44^ ^]^

In addition, it has been observed that phenolic compounds can also increase levels of endogenous antioxidants as reduced glutathione (GSH). Hepatic GSH levels were increased in animals administered resveratrol in comparison to control group.^[^ ^41^, ^45^, ^46^ ^]^ Regarding the effect of phenolic‐rich extracts or foods, they can also exert antioxidant effects by different mechanisms including a decrease of hepatic ROS levels^[^ ^47^ ^]^ and *aorta Nox4* mRNA expression and an increase of hepatic GSH levels,^[^ ^47^, ^48^, ^49^, ^50^ ^]^ plasma or kidney SOD activity^[^ ^51^ ^]^ and expression of aortic *Sod2* ^[^ ^52^ ^]^ in hypertensive animals respect to control animals. Moreover, it has been observed a restoring of the activity of SOD, CAT,^[^ ^53^, ^54^ ^]^ GPx,^[^ ^54^, ^55^ ^]^ glutathione reductase, or glutathione S‐transferase^[^ ^55^ ^]^ in other animals models suffering oxidative stress.

Moreover, ROS production represents the main cause of endothelial dysfunction. Vascular endothelium regulates vascular tone and exerts finely tuned control over cardiovascular and metabolic homeostasis. Endothelial cells secrete and absorb vasoactive and vasoconstrictor compounds to regulate vascular tissue.^[^ ^56^ ^]^ The reduction of ROS levels by phenolic compounds leads to an improvement of endothelial dysfunction due to a decrease in the oxidation of the endothelial vasodilator nitric oxide (NO), which results in an increase of the NO availability.^[^ ^52^ ^]^ Endothelial NO is synthesized through the oxidation of l‐arginine to l‐citrulline in a reaction catalyzed by the endothelial Ca^2+^‐dependent constitutive isoform of NO synthase (eNOS).^[^ ^57^ ^]^ In addition, ROS can also produce excessive oxidation and depletion of the eNOS cofactor tetrahydrobiopterin (BH_4_)^[^ ^58^ ^]^ favoring eNOS uncoupling.^[^ ^59^ ^]^ Thus, eNOS starts to generate superoxide instead of NO, reducing NO production and increasing oxidative stress.^[^ ^60^ ^]^ Human intervention studies have reported an improvement of NO‐dependent flow‐mediated dilation (FMD) after the intake of phenolic compounds, indicating that these compounds can improve endothelial dysfunction.^[^ ^8^, ^61^ ^]^ Indeed, FMD is considered as an accepted technique to quantify endothelial function, which has been inversely associated with future CVD events.^[^ ^62^ ^]^ In addition, some flavonoids or their derivatives (i.e., diosmin, hesperidin, rutin, and quercetin) are widely used as pharmaceutical agents for their vasoprotective properties (i.e., Daflon 500 and Venorutom).^[^ ^63^, ^64^ ^]^

High levels of ROS have also been associated with an increase of lipid peroxidation.^[^ ^65^ ^]^ Malondialdehyde (MDA), a secondary product produced by decomposition of arachidonic acid and larger PUFAs,^[^ ^66^ ^]^ is involved in different CVD risk factors, interacting with free amino acids or proteins, generating Schiff‐base adducts.^[^ ^66^, ^67^ ^]^ These adducts have been shown to exert proinflammatory and atherogenic effects.^[^ ^65^ ^]^ Moreover, MDA contributes to endothelial dysfunction producing a reduction on eNOS activity and expression, and therefore reducing NO availability.^[^ ^68^ ^]^ It has been reported a reduction on MDA levels in spontaneously hypertensive rats administered red wine pomace extract,^[^ ^52^ ^]^ wine lees liquid fraction,^[^ ^47^ ^]^ grape skin extract,^[^ ^51^ ^]^ or GSPE.^[^ ^50^ ^]^

In addition to reducing ROS and MDA levels, there is evidence that phenolic compounds can increase NO availability by acting directly in NO production pathway.^[^ ^69^, ^70^, ^71^, ^72^ ^]^ An increase of NO availability could be produced by a restoration of endothelial NO levels to healthy values. In this regard, some studies have observed an increase in plasma NO levels after consumption of different phenolic‐rich extracts or foods respect to nontreated group.^[^ ^37^, ^47^, ^73^ ^]^ In this regard, an endothelium‐dependent NO‐mediated vasodilation was observed in pig coronary arterial rings treated with chokeberry and bilberry extracts.^[^ ^74^ ^]^ Similar mechanism was observed in a double‐blind study, in which healthy volunteers drank a flavanol‐rich cocoa during 5 days.^[^ ^75^ ^]^ Moreover, studies carried out in hypertensive rats with compromised NO production pathway have also demonstrated the role of NO in the antihypertensive effect of a wine lees liquid fraction^[^ ^76^ ^]^ or GSPE.^[^ ^69^, ^77^ ^]^ This increase in NO can be mediated trough an upregulation of *e* *NOS*.^[^ ^78^, ^79^ ^]^ In this regard, an upregulation of *eNOS* mRNA expression was observed in endothelial cells treated with resveratrol,^[^ ^80^, ^81^, ^82^ ^]^ pomegranate juice,^[^ ^83^ ^]^ or pomegranate fruit extract.^[^ ^84^ ^]^ Similar effects were also observed in rats administered resveratrol,^[^ ^37^, ^78^ ^]^ pomegranate extract,^[^ ^85^ ^]^ alibernet red wine,^[^ ^86^ ^]^ GSPE,^[^ ^49^ ^]^ or a wine lees liquid fraction.^[^ ^76^ ^]^ Aditionally, phenolic compounds such as resveratrol, tannic acid, quercetin, rutin, and seapolynol can activate the eNOS‐promoter transcription factor *Krüpple like factor 2* (*KLF2*) gene expression.^[^ ^87^, ^88^ ^]^ Moreover, KLF2 can regulate eNOS uncoupling via the nuclear factor erythroid 2‐related factor 2/HO‐1 pathway in endothelial cells under hypoxia and reoxigeneration.^[^ ^89^ ^]^

Another molecular mechanism that could explain the vasodilator effect of phenolic compounds via NO is the modulation of eNOS activity. In this regard, it has been observed an enhanced eNOS activity in human umbilical vein endothelial cells treated with quercetin.^[^ ^79^ ^]^ Moreover, a restoration of cardiac eNOS activity was observed in fructose‐fed rats, an experimental model of insulin resistance syndrome with endothelial dysfunction, after consumption of resveratrol for 45 days.^[^ ^90^ ^]^ One of the mechanisms by which phenolic compounds can modulate eNOS activity is by acting on SIRT1 pathway. SIRT1 activates eNOS through its deacetylation and influences *eNOS* transcription, leading to enhancement of NO production.^[^ ^91^, ^92^ ^]^ Moreover, SIRT1 can inhibit endothelial apoptosis and improve vascular endothelial function exerting antiatherosclerotic effects.^[^ ^93^ ^]^Thus, the BP‐lowering effects of a wine lees liquid fraction^[^ ^76^ ^]^ or GSPE^[^ ^94^ ^]^ were partially or totally abolished when hypertensive rats were treated with the SIRT1 inhibitor, sirtinol. The effect of these phenolic‐rich extracts was attributed to an upregulation of endothelial *Sirt1* expression since animals administered extracts showed increased levels of aortic *Sirt1* expression.^[^ ^49^, ^76^, ^94^ ^]^ Changes in *SIRT1* expression were also observed in peripheral blood mononuclear cells of volunteers consuming a nonalcoholic red wine extract for eight weeks.^[^ ^95^ ^]^ Other studies have shown that phenolic compounds such as curcumin, quercetin, luteolin, and resveratrol can be SIRT1 activators,^[^ ^96^ ^]^ providing other mechanism by which phenolic compounds can modulate SIRT1 pathway. Therefore, phenolic compounds may also exert their vasoprotective effects increasing the NO production by acting on SIRT1, increasing the synthesis, and/or activity of this enzyme. It is worthy to mention that SIRT1 can downregulate *NOX4*,^[^ ^97^ ^]^ which may produce an improvement in NO availability.

In addition to NO, phenolic compounds can regulate the production of other endothelial factors such as prostaglandin I2 (PGI2). PGI2, an endothelial factor produced from arachidonic acid by mediation of cyclooxygenase isomer 2 (COX‐2) and prostacyclin synthase,^[^ ^98^ ^]^ is a potent inhibitor of platelet aggregation^[^ ^99^ ^]^ and a vasodilator under NO deficit.^[^ ^100^ ^]^ The antihypertensive effect of GSPE and a wine lees liquid fraction was partially mediated by PGI2.^[^ ^69^, ^76^, ^77^ ^]^ Several studies showed an increase in PGI2 or prostaglandinf1 alpha levels, a stable metabolite of PGI2, in human aortic endothelial cells after treatment with chocolate procyanidins ^[^ ^101^ ^]^ and in plasma of rats^[^ ^69^, ^102^ ^]^ after consumption of flavanol‐rich extracts. Finally, it has been reported that phenolic compounds can also modulate the production of the endothelial vasoconstrictor endothelin 1 (ET‐1).^[^ ^94^ ^]^ *ET1* expression is downregulated by KLF‐2.^[^ ^103^ ^]^ Phenolic compounds of red wine produced a reduction of plasma ET‐1 levels in hypertensive volunteers consuming a single dose of 8.1 dL of wine.^[^ ^104^ ^]^ Similar effects were observed in hypertensive rats administered GSPE.^[^ ^77^ ^]^ In addition, aortic *Et1* mRNA expression was downregulated in animal administered GSPE and a wine lees liquid fraction.^[^ ^76^ ^]^ Moreover, phenolic compounds can exert their beneficial effects by different mechanisms such as acting on the renin‐angiotensin‐aldosterone system, which is one of the main blood pressure regulatory mechanisms. More concretely, the main target in this system is angiotensin‐converting enzyme (ACE). In vivo ACE inhibition has been demonstrated to be effective in reducing BP, since this enzyme synthetizes the vasoconstrictor angiotensin II (ANGII).^[^ ^105^ ^]^ ANGII is a major contributor to hypertension via its central, vascular, and renal effects. Moreover, ANGII is also able to activate NOX‐derived ROS production.^[^ ^106^, ^107^ ^]^ In vitro studies have demonstrated ACE inhibitory properties of different phenolic compounds.^[^ ^108^, ^109^ ^]^ Moreover, phenolic compounds such as quercetin can inhibit ACE in hypertensive animals.^[^ ^42^ ^]^

## 3 Biological Rhythms Influence on Cardiovascular System

### 3.1 The Circadian Clock

Organisms adapt their behavior and physiology to environmental light/dark cycles resulting from the rotation of the Earth. In mammals, these rhythms are regulated by central and peripheral clocks.^[^ ^110^ ^]^ The central clock is located in the suprachiasmatic nucleus of the hypothalamus and is mainly synchronized by light, the most important environmental cue or “zeitgebers”^[^ ^110^ ^]^ Peripheral clocks are present in almost all tissues and are set by signals from the central clock in a hierarchical organization.^[^ ^111^ ^]^ These peripheral oscillators are also entrained by other zeitgebers such as exercise and nutrition.^[^ ^110^ ^]^

The circadian intracellular machinery of molecular clocks is made up of two master transcription factors: circadian locomotor output cycles kaput (CLOCK) and brain and muscle arnt‐like protein‐1 (BMAL1). After dimerization, they induce expression of genes including their negative regulators *Period* (*PER1*, *PER2*, and *PER3*) and *Cryptochrome* (*CRY1* and *CRY2*). PER and CRY are accumulated in the cytoplasm and once critical concentration is reached, they translocate to the nucleus promoting the dissociation of CLOCK:BMAL1 heterodimer and their own transcription. CLOCK:BMAL1 heterodimer also induces the expression of the nuclear receptors *RER‐ERBα* and *RER‐ERBβ*, inhibitors of *BMAL1* expression. These transcriptional/translational feedback loops show a periodicity of 24 h.^[^ ^112^ ^]^ Phosphorylation and ubiquitylation‐mediated degradation of PER and CRY regulates the timing of circadian cycles. A post‐transcriptional control of circadian machinery mediated by phosphorylation and acetylation has also been reported. Moreover, CLOCK has been shown to present histone acetyltransferase activity, enabling cycles of acetylation and deacetylation of several proteins of the core clock apparatus.^[^ ^113^ ^]^ The latter activity also involves SIRT1, which in turn regulates circadian rhythms by deacetylating both histones and nonhistone proteins such as BMAL1 and PER2. Moreover, nuclear receptors such as REV‐ERBα, retinoic acid orphan receptor alpha and beta (RORα and RORβ, respectively), peroxisome proliferator—activated receptor‐alpha (PPARα) and peroxisome proliferator—activated receptor‐gamma (PPARγ) coactivator 1‐α (PGC1α) constitute a short feedback loop controlling *BMAL1* transcription. In addition, CLOCK and BMAL1 also induce the expression of some of these nuclear receptors such as *REV‐ERBα* and *PPARα*.^[^ ^114^ ^]^ Moreover, circadian clock gene expression in the pituitary gland is modulated by photoperiod via melatonin signal leading to changes in the relative phase of PER and *CRY* gene expression and generating a long‐term photoperiodic response (circannual rhythms).^[^ ^115^ ^]^

### 3.2 Molecular Basis of Circadian Control of Cardiometabolic Factors

The circadian clock controls many key proteins involved in the regulation of important metabolic pathways such as the expression or activity of rate‐limiting enzymes.^[^ ^116^ ^]^ For example, the regulation of nicotinamide phosphoribosyl transferase (NAMPT), the rate‐limiting enzyme of the nicotinamide adenine dinucleotide (NAD) pathway, oscillates in a circadian manner leading to circadian rhythmicity of NAD^+^ levels.^[^ ^117^ ^]^ In turn, NAD^+^ is needed for SIRT1 deacetylase activity, which is responsible for the regulation of important genes involved in metabolic pathways, linking cellular energy metabolism and gene expression. Therefore, NAD^+^ integrates circadian rhythms and nutrient sensing pathways through SIRT1 functionality. In addition, SIRT1 also regulates several gluconeogenic genes through the activation of *Forkhead box O1* (*FOXO1*) and *PGC1α*.^[^ ^118^ ^]^ In addition, adenosine monophosphate kinase (AMPK), which is activated when the cellular energy status is low, also modulates the circadian cycle via phosphorylation of CRY1 and casein kinases CKIe. When the AMP/ATP ratio is high, this enzyme activates catabolic processes to obtain ATP and deactivates ATP‐consuming processes. Interestingly, the activity of AMPK was found to be rhythmic in the liver, hypothalamus, and fibroblasts of mice.^[^ ^119^ ^]^ As AMPK activation also leads to an increase in NAD^+^ levels,^[^ ^120^ ^]^ AMPK could also modulate circadian gene expression indirectly via SIRT1 activation.

As mentioned above, clock genes modulate nuclear receptors, and these are also involved in the regulation of important metabolic pathways. Thus, REV‐ERBα regulates lipid metabolism and adipogenesis, and PPARα controls fatty acid oxidation and apolipoprotein synthesis, demonstrating an interaction between nuclear receptors, metabolism, and circadian clock.^[^ ^114^ ^]^ PGC1α also regulates lipid metabolism and it is considered a critical metabolic regulator in many vital organs.^[^ ^121^ ^]^ Indeed, circulating lipids follow circadian rhythms increasing during the active period and falling during the rest period.^[^ ^122^ ^]^ Interestingly, enterocytes rhythmically express molecular clock genes, and lipid absorption efficiency by these cells is high during the active phase and low during the rest phase.^[^ ^123^ ^]^ However, *Clock^Δ19/Δ19^* mice lack the circadian pattern of enterocyte gene expression and lipid absorption, displaying three‐fold more blood cholesterol than wild‐type mice.^[^ ^124^ ^]^ In addition, macrophages from these animals took up more oxidized lipids and were defective in cholesterol efflux. In this regard, molecular studies showed that CLOCK regulates *ABCA1* expression and cholesterol efflux in macrophages. Hepatic lipogenesis also displays circadian fluctuations. For instance, genes mediating hepatic triglyceride and cholesterol synthesis including fatty acid synthase, SREBP‐1c, acetyl‐CoA synthase, acetyl co‐A carboxylase, and 3‐hydroxy‐3‐methylglutaryl‐coenzyme A show circadian expression rhythmicity in animals under high‐fat diet.^[^ ^125^ ^]^ In addition, it has been recently reported that global and hepatic *Bmal1* knockout mice have an impaired cholesterol metabolism and present abnormal aortic atherosclerotic lesions.^[^ ^126^ ^]^

Moreover, PER2 interacts with several nuclear receptors including PPARg, estrogen receptors alpha, PPARα, REV‐ERBα, hepatocyte nuclear factor 4 alpha, thyroid hormone receptor‐α, nuclear receptor related 1, and RORα. These interactions modulate the expression of core clock genes such as *BMAL1* and influence several metabolic pathways such as lipid and glycogen metabolism. In this regard, PER2 inhibits the recruitment of PPARg to its target promoters in white adipose tissue (WAT).^[^ ^127^ ^]^

Furthermore, macrophage and monocyte C‐C motif chemokine ligand 2 (CCL2), which is a critical chemokine in atherogenesis, presents a clear circadian rhythmicity both in their expression and production.^[^ ^128^ ^]^ In this regard, it has been shown that circadian machinery mediates macrophage functionality and up to 8% of the macrophage transcriptome fluctuates over 24 h.^[^ ^129^ ^]^ Moreover, the formation of the nod‐like receptor protein 3 (NLRP3) inflammasome and its constituent cytokine IL‐1β are transcriptionally controlled by REV‐ERB.^[^ ^130^ ^]^ Additionally, it has been shown that the pharmacological targeting of REV‐ERB downregulates the adverse inflammasome activity and improves outcomes after myocardial infarction.^[^ ^131^ ^]^ In this regard, the cardiomyocyte‐specific loss of BMAL1 influences inflammation and modulates CVD outcomes.^[^ ^132^ ^]^

BP and cardiac frequency, other important cardiometabolic factors, also show a circadian rhythm characterized by a decrease during the rest phase and an increase during awakening and the beginning of the activity, matching with the maximum cortisol peak in blood.^[^ ^133^ ^]^ Corticotropin‐releasing hormone, the adrenocorticotropic hormone, and glucocorticoids are involved in BP and cardiac frequency modulation and have also shown circadian rhythmicity.^[^ ^133^, ^134^ ^]^ In addition, different endothelial factors involved in the regulation of BP and vascular tone, follow also circadian rhythmicity. Thus, the expression of different enzymes involved in the production of the endothelial vasodilator NO, including of the eNOS, is under control of the circadian clock and peaks during the active‐period.^[^ ^135^ ^]^ Additionally, GTP cyclohydrolase‐1 and dihydrofolate reductase, involved in the production of eNOS BH_4_ cofactor, are both under direct control of the circadian clock and present a peak during the active‐period.^[^ ^136^ ^]^ In addition, SIRT1 activates eNOS through its deacetylation and influences in the eNOS transcription, leading to enhanced NO production.^[^ ^91^, ^92^ ^]^ Moreover, NOX1 and NOX4, involved in the endothelial production of ROS, show a strong oscillation along the day.^[^ ^137^ ^]^

### 3.3 Circadian Disruption and Cardiometabolic Diseases

Changes in the light‐dark cycles and constant exposure to artificial light induce misalignment of the clocks that is associated with alteration in the expressions of genes and synthesis of proteins, conditioning the onset of several diseases.^[^ ^138^, ^139^ ^]^ Concerning cardiovascular health, it has been shown that rhythm disruption alters normal vascular function, BP, and increases the probability of suffering atherosclerosis, stroke, and related CVD.^[^ ^140^ ^]^ In this regard, disruption of the rhythms alters a cohort of multiple entangled biological processes, which in turn can also induce a higher prevalence of strokes and other cardiovascular events at any time.^[^ ^141^ ^]^

Shift‐workers are especially sensitive to disruption of circadian rhythms due to alterations in their natural light‐dark schedule. This alteration is reflected in their BP levels which, in contrast with daily workers, are lower during the morning than in the afternoon or evening.^[^ ^142^ ^]^ Accordingly, different meta‐analysis involving shift workers concluded that the risk of CVD was 17% higher among this group than day workers^[^ ^143^ ^]^ and that shift work contributes to the onset of obesity and related comorbidities.^[^ ^144^ ^]^

Sleep disorders are also associated to circadian deregulation, contributing to the development of inflammation, obesity, diabetes, and CVD.^[^ ^145^ ^]^ For instance, it has been described that sleep curtailment reduces the expression of key clock circadian genes. This fact is associated with alteration of the normal function of the beta cells, potentially increasing the development of type 2 diabetes.^[^ ^146^ ^]^ Moreover, enlarged sleep duration (more than 8 h) as well as shortened sleep (less than 7 h) are associated with atherosclerosis, coronary arteries’ calcification, and stroke. An imbalance in the hormonal levels that leads to diabetes and obesity are believed to be some of the underlying mechanisms linked to the onset of this plethora of CVD issues.^[^ ^147^ ^]^

Another factor involved in disruption of biological rhythms is lifestyle, including sedentariness, level of physical activity, and social jetlag. For example, sedentary work is linked with a higher prevalence of CVD and negative metabolic health outcomes than those works that have a higher level of physical activity.^[^ ^148^ ^]^ In addition, exercise is able to reset rhythmicity of altered clock genes in several tissues, acting as external zeitgeber and can be useful for the treatment or alleviation of metabolic diseases.^[^ ^149^ ^]^ Regarding social jetlag, one of the main disruptors in the actual society, it is produced by the misalignment between the biological clock and the resulting social clock derived from social activities, work and, other constraints.^[^ ^150^ ^]^ Social jetlag is associated in a positive manner with CVD and endocrine disorders in healthy subjects. Thus, social jet lag produces increased levels of triglycerides, insulin resistance, and adiposity and decreased HDL cholesterol.^[^ ^151^ ^]^ Therefore, synchronizing biological and social clock could contribute to the management of several diseases.

Finally, nutrition is one of the most important factors that contribute to the disruption of biological rhythms. Dietary patterns (including timing, number of meals, restrictions, and diet composition) act as zeitgebers, and can cause a misalignment between the central and the peripheral clocks that results in the development of metabolic disorders.^[^ ^152^ ^]^ It is suggested that the effects of diet on the clock genes are reversible and that nutritional challenges, including phytochemicals such as phenolic compounds, may act resynchronizing these clocks.^[^ ^153^ ^]^

## 4 Circadian Influence on the Cardiometabolic Effects of Phenolic Compounds

In addition to their effects on inflammation, oxidative stress and endothelial function, phenolic compounds may prevent CVD by modulating the circadian clock. In this regard, we have recently discussed that their extensive activity could be explained by their potential to stimulate homeostasis through interactions with the biological clock system.^[^ ^13^ ^]^ Hence, biological rhythms have been shown to impact in phenolic compound absorption and metabolism, influencing their bioactivity and, at the same time, phenolic compounds may modulate clock genes and related signaling pathways. This is in accordance with the xenohormesis hypothesis.

Several studies have shown different effects of phenolic compounds depending on the administration time. For example, antioxidant effects as well as changes of the hepatic and central clock gene expression and in the levels of important metabolites including plasma glucose, NAMPT, NAD^+^, and melatonin among others, were observed depending on the administration time of different phenolic compounds including resveratrol, or extracts such as GSPE in both humans and animal studies.^[^ ^13^ ^]^ In this context, the participation of clock genes in the cardioprotective effects of proanthocyanidins has been studied by our group. Thus, GSPE modulated both central and peripheral biological rhythms altering the expression of hypothalamic clock genes such as *Bmal1* and *Nampt*.^[^ ^154^ ^]^ Furthermore, different doses of these compounds administered to diet‐induced obese rats during 4 weeks exerted a positive dose‐dependent modulation of some components of the peripheral clock in liver, gut and WAT, including *Bmal1*, *Nampt*, *Sirt1*, and NAD^+^.^[^ ^155^, ^156^, ^157^ ^]^ The modulation of both *Sirt1* expression and levels of its cofactor NAD^+^ suggest the participation of biological rhythms in the cardioprotective effects of phenolic compounds though this deacetylase, key in the cardiovascular system.

In addition, we have demonstrated that not only circadian rhythms can impact on phenolic compound bioactivity, but also seasonal rhythms may play a key role. Thus, exposure of Fischer 344 rats to different photoperiods significantly impacted red grape polyphenol bioavailability.^[^ ^158^ ^]^ Significant differences on parameters related to hypothalamic appetite signaling pathways such as leptin sensitivity and *proopiomelanocortin* (*Pomc*) expression were also observed when administering phenolic‐rich fruits (cherry and grape).^[^ ^159^ ^]^ In addition, lipid and glucose homeostasis was altered following intake of fruits out‐of‐season in both standard and cafeteria diet‐fed rats.^[^ ^160^, ^161^ ^]^ In contrast, consumption of tomatoes in‐season improved antioxidant biomarkers, thus reducing oxidative stress values.^[^ ^162^ ^]^ Hence, the physiological and molecular changes elicited by seasonal fruit consumption could be partially explained by the modulation of the mammalian clock system. Actually, both cherry and orange consumption have been correlated with the modulation of mRNA levels of peripheral clock‐related genes like *nuclear receptor subfamily 1 group D member 1* (*Nr1d1)* in skeletal muscle, and *Per2* or *Cry1* in the liver and WAT of healthy rats when they are consumed out of season.^[^ ^159^, ^161^, ^163^ ^]^ Other beneficial effects on cardiovascular risk factors of plant phenols have also been linked to the modulation of central and peripheral clock system. For example, the amelioration of obese‐related metabolic alterations exerted by epigallocatechin‐3‐gallate seems to be linked with the regulation of the rhythmic expression of circadian clock genes such as *Clock*, *Bmal1*, and *Cry1* in C57BL/6J mice.^[^ ^164^ ^]^ In addition, resveratrol restored the circadian synchrony of lipid metabolism altered by a high‐fat diet modifying the rhythmic expression of both clock genes and clock‐controlled lipogenic genes in WAT.^[^ ^165^, ^166^ ^]^

As stated above, the interaction between phenolic compounds and biological rhythms is bidirectional so that these compounds may also modulate expression of clock genes.^[^ ^13^ ^]^ Thus, flavonoids such as nobiletin and hesperidin enhanced the circadian rhythm amplitude in obese mice and in a fruit‐fly model respectively, while others such as myricetin reduced it in Sprague‐Daw rats.^[^ ^167^ ^]^

In addition to flavonoids, other phenolic compounds with proved cardioprotective properties, such as phenolic acids or resveratrol have also shown capability to modulate circadian rhythms. Thus, vanillic acid enhanced circadian amplitude while cinnamic acid reduced it in rats and mice studies, respectively.^[^ ^168^, ^169^ ^]^ Resveratrol has been also shown to modulate the expression of clock genes such as *Sirt1*, *Per1*, *Per2*, *Bmal1*, and *REV‐ERBα* in different animal studies.^[^ ^167^, ^170^ ^]^

In addition, gut microbiota may be playing a key role in this interaction between phenolic compounds and biological rhythms. Thus, both circadian and seasonal rhythms have been shown to promote gut microbiota oscillations in relative abundance and function, mostly driven by changes in diet and feeding patterns.^[^ ^171^, ^172^ ^]^ This may have a significant impact on phenolic compounds cardioprotective effects as gut microbiota is significantly involved in phenolic compounds metabolism and, therefore, its alteration may lead to the production of different metabolites with different bioactivities.^[^ ^173^ ^]^ Indeed, glycosylated forms and high molecular weight polymers of dietary phenolic compounds are metabolized by gut bacteria to lower molecular weight phenolic compounds that can be further metabolized by these bacteria. For example, isoflavone is metabolized by gut microbiota to equol, a compound that have been shown to reduce atherosclerosis lesions in the aorta, to reduce triglycerides, total cholesterol, and LDL cholesterol, and to increased HDL cholesterol. Ellagitannins and ellagic acid also suffer extensive metabolism by the gut microbiota leading to the production of urolothins, which are better absorbed and have been shown to exert several beneficial effects including cardioprotective, anti‐inflammatory, and antioxidant effects.^[^ ^174^, ^175^ ^]^ Resveratrol is also modified by gut microbiota producing other metabolites such as piceid, which has shown antioxidant and anti‐inflammatory activities. Anthocyanins are also degraded by gut microbiota to metabolites such as protocatechuic acid which have shown different cardioprotective effects.^[^ ^176^ ^]^ At the same time, phenolic compounds may also modulate gut microbiota composition. In fact, it has been proposed that polyphenols that are not absorbed in the upper intestinal tract may exert their beneficial effects primarily acting through gut bacteria modulation.^[^ ^177^, ^178^, ^179^ ^]^ Thus, gut microbiota have been largely recognized for its importance in modulating several metabolic and physiologic processes including those related to CDV.^[^ ^180^ ^]^ Indeed, gut microbiota affects lipid absorption and bile acid turnover, which have a strong influence on cholesterol homeostasis,^[^ ^181^ ^]^ impacts short‐chain fatty acids production and the storage of fat, and interacts with hepatic genes.^[^ ^182^ ^]^ Most of these effects are transduced by microbiota signals like Toll‐like receptors (TLRs)^[^ ^183^ ^]^ and the immune system.^[^ ^184^ ^]^ In addition, through the gut‐brain axis, gut bacteria affect the hypothalamic pituitary adrenal axis and the production of corticosteroids involved in endothelial tone.^[^ ^185^ ^]^ Moreover, gut microbiota has also been proposed to act as a transducer of dietary cues to regulate host circadian rhythms. Indeed, changes in gut microbiota under different diets have been shown to alter central and peripheral host circadian clock functions.^[^ ^186^, ^187^ ^]^ Therefore, this bidirectional interaction between gut microbiota and phenolic compounds may be playing a pivotal role on their biological rhythms modulation effects.

## 5 Future Perspectives

Circadian and seasonal rhythms should be considered in the study of the functionality of phytochemicals such as phenolic compounds. Unfortunately, there are not many studies available and most have been focused on food rich in melatonin, a key element of the internal clock, or its precursors tryptophan and serotonin, as reviewed in our recent work.^[^ ^13^ ^]^ Further studies to elucidate the potential mechanisms involved in the polyphenols–biological rhythms interaction are needed. In addition, the effect of phenolic compounds on biological rhythms can be also mediated by microRNA. In this context recent unpublished results of our group shows that the activity of proanthocyanidins as modulators of hepatic clock genes is mediated by miRNAs.

## Conflict of Interest

The authors declare no conflict of interest.
